# Note from the editors: *Eurosurveillance*, 1996 to 2021 – 25 years of public health impact

**DOI:** 10.2807/1560-7917.ES.2021.26.18.2105061

**Published:** 2021-05-06

**Authors:** Eurosurveillance editorial team

**Affiliations:** 1 European Centre for Disease Prevention and Control (ECDC), Stockholm, Sweden

Over the past 25 years, *Eurosurveillance* has served the public health and communicable disease communities in Europe and beyond by publishing authoritative, sound evidence for immediate and long-term public health action. To mark the occasion, we have compiled a collection of 25 articles published since the journal’s inception: *Eurosurveillance,* 1996 to 2021: 25 years of public health impact.

Thanks to valuable input from our editorial board, current and former colleagues, and other peers, the selection of articles provides examples—based on personal experiences—of the impactful role the journal has played in different countries, epidemics and infectious disease areas, with particular focus on the past 10 years [1,2]. Among these are articles that have supported public health experts to advocate for the introduction of reimbursement for TBE vaccination among children in Slovenia [3], guided the set-up of online and harmonised influenza reporting from the Czech Republic to the European surveillance network [4] and served as evidence for guidance on the prevention of sexual transmission of Zika virus by the World Health Organization [5,6]. The ongoing COVID-19 pandemic is also represented in this anniversary selection, including the first publication describing a diagnostic workflow used to detect SARS-CoV-2 [7].

Anniversaries are a time of reflection, but they also inspire us to look forward. This collection underscores the impact that publications in *Eurosurveillance* have had on public health practice, policy and science, while also reminding us of the importance of continuing our efforts to support evidence-based public health practices and policies, as well as equitable access to trustworthy information for communicable disease prevention and control.
